# Rural Appalachian Women Will Suffer Disproportionately if Attempts to Further Restrict Emergency Contraception are Successful

**DOI:** 10.12023/jah.0501.02

**Published:** 2023-04-01

**Authors:** Amie M. Ashcraft, Sarah Dotson, Sara Farjo, Courtney S. Pilkerton, Pamela J. Murray

**Affiliations:** West Virginia University, amashcraft@hsc.wvu.edu; West Virginia University; West Virginia University; West Virginia University; Boston Children’s Hospital

**Keywords:** Appalachia, abortion, contraception, emergency contraception, levonorgestrel, rural health disparities

## Abstract

The removal of federal abortion protection has incited fear that restrictions on contraception may be next. Many states now imposing abortion restrictions and bans are in the South and Appalachian Regions of the U.S., where rates of unplanned pregnancy and poor health outcomes are already disproportionately high. Numerous studies have documented variable access to levonorgestrel EC (LNG EC) in community pharmacies, with particularly low rates of access at independent pharmacies that are more likely to be located in rural communities than chain pharmacies. Since the overturn of *Roe v. Wade*, some large chain pharmacies and online retailers are restricting the purchase of LNG EC, limiting its availability. Some legislators and activists are calling for a ban on EC based on a misunderstanding about its mechanism of action, equating it with abortion. At a time when access to the full range of contraceptive options is more critical than ever, already limited access to LNG EC is worsening. Extensive data on LNG EC availability in 509 pharmacies and 400 health clinics across West Virginia, contextualized with socioeconomic demographics, illustrate existing disparities in LNG EC access.

## INTRODUCTION

The U.S. Supreme Court’s decision in *Dobbs v. Jackson Women’s Health Organization* overturned *Roe v. Wade* and removed federal protection for abortion. This ruling has incited fear that access to contraception may be in danger. According to data from the Guttmacher Institute, 26 states are likely to ban abortion after the overturn of *Roe*.^1^ At the time of this writing, 14 states have banned or restricted abortion.^2^ Many of these states are in the South and Appalachian Regions, which have higher rates of unplanned pregnancy and poorer health outcomes for women.^3^–^4^ Increasing restrictions on abortion means a full range of available, accessible contraceptive options, including emergency contraception (EC), is more important than ever. Unfortunately, with the loss of constitutional protection for reproductive rights, some states have begun targeting EC.^5^

### Plan B May Be Next

Levonorgestrel emergency contraception (LNG EC), commonly known by the brand name Plan B®, has been undermined by misinformation and misconceptions about its mechanism of action since the Food & Drug Administration’s (FDA) first review in 2006. A physician on the scientific review panel argued there was a chance the medication could prevent the implantation of a fertilized egg.^6^–^7^ Although there was no evidence to support this statement, the FDA required the drug company to list this possibility on the medication. Mislabeling LNG EC as an abortifacient—a drug that causes an abortion—set the stage for “conscientious objection,” or refusal of its provision based on moral or religious grounds.^8^ Many activists who believe life begins with fertilization have used the mislabeling to argue for a ban on EC. In the last year alone, several legislators have publicly equated or introduced legislation equating EC with abortion.^9^ This labeling was only just changed by the U.S. FDA in December 2022^10^ to be consistent with accumulated evidence that LNG EC does not prevent a fertilized egg from implantation.^11^–^12^

Purchase restrictions for LNG EC are currently occurring at Walmart, Rite Aid, and Amazon as women “stock up” amid new abortion bans and restrictions.^13^–^14^ Even before the overturn of *Roe*, there were significant and pervasive disparities in access to LNG EC. Several studies have shown low stocking rates across chain and independent community pharmacies.^15^–^23^ Evidence also indicates barriers to access—such as not stocking LNG EC and point-of-sale requirements beyond U.S.-government-set regulations—are highest at independent pharmacies and pharmacies located within lower-resource communities.^15^–^17^,^23^–^26^ Both independent pharmacies and poor communities are more heavily concentrated in rural regions of the U.S., such as Appalachia.^27^–^30^

### Women in Rural Communities Will Suffer Disproportionately

Barriers to reproductive healthcare access are increasing for women across the U.S., with most concentrated in rural communities.^27^,^31^ Many disadvantages exist: absence of comprehensive sex education, long distances to health clinics, lack of transportation, high cost of gasoline, limited clinic and pharmacy hours of operation, high healthcare costs, and high rates of uninsured and underinsured. These factors converge with increasing poverty rates and continued small-town clinic closures to increase the likelihood of unintended pregnancies in rural areas by limiting women’s access to reproductive healthcare.^32^–^33^ Without the ability to fully control if and when they become parents, rural women continue to face adversity due to disparities in reproductive healthcare.

There is a lack of access to high quality maternal health services in rural communities due to hospital and obstetric department closures, diminished public health and family planning infrastructure, workforce shortages, and socioeconomic access to care challenges.^34^ Nearly half of all rural counties in the U.S. do not have a hospital with obstetric services.^35^ Subsequently, fewer than 50% of rural women have access to perinatal services within a 30-minute drive of their homes, and more than 10% of rural women drive 100 miles or more (ACOG, 2014).^27^ Limited maternal healthcare access and delays in care can lead to a host of negative fetal and maternal health outcomes^36^–^40^ and contribute to the cycle of poverty.

### Section IV: The Case of WV

As the only state with borders located completely within Appalachia, 64% of West Virginia (WV) is classified as rural.^41^ WV has the fourth-highest poverty rate,^42^ the eighth-highest teen birth rate,^43^ and ranks at or near the bottom of states on factors increasing the likelihood of poor maternal and child health outcomes, including poverty, unemployment, lack of healthcare access, obesity, chronic disease, tobacco use, and substance use disorder.^44^–^46^

Even prior to the overturn of *Roe*, women in WV had restricted reproductive options, with only one clinic in the state providing abortions since 2017.^47^ We conducted a mystery caller study with WV community pharmacies to assess LNG EC availability and found that fewer than half (48.9%) reported having LNG EC in stock, with dramatic differences between the stocking rates of chain pharmacies (76.3%) and independent pharmacies (14.6%).^15^ Even when pharmacies reported having LNG EC in stock, young women calling about requirements for purchase were often told incorrectly that there was a minimum age requirement or that a prescription, identification, or parental consent were required.^15^,^48^ Misinformation about sales requirements and timing for effectiveness was high.

Compared to pharmacies, even fewer health clinics in WV reported having LNG EC in stock. We also contacted health clinics of all types across the state to assess LNG EC availability, and it was reported to be in stock at 43.2% of clinics (see Tables 1 & 2). Table 1 displays a breakdown of the types of health clinics contacted by our team (n = 404), and Table 2 displays the proportion of clinic types who reported having LNG EC in stock. County health departments reported having LNG EC in stock most often (47.1%). Rural Health Clinics, Federally Qualified Health Centers, and free clinics were the least likely to report having it in stock (17.9%, 17.1%, and 10.0%, respectively). Clinic staff frequently referred female callers in our study to their local pharmacy to obtain LNG EC, likely not knowing how difficult it is to access in WV pharmacies.

**Figure 1:**
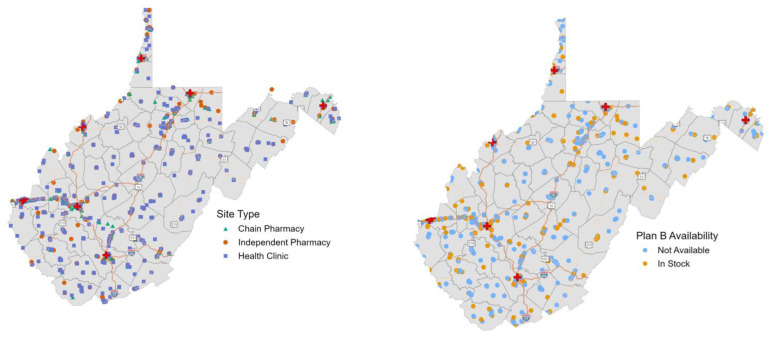
Site distribution and reported Plan B availability by site NOTE: Health clinic data were previously unpublished; pharmacy data used for mapping and methodology for health clinic data collection are described in Ashcraft et al., 2020.^15^

**Figure 2:**
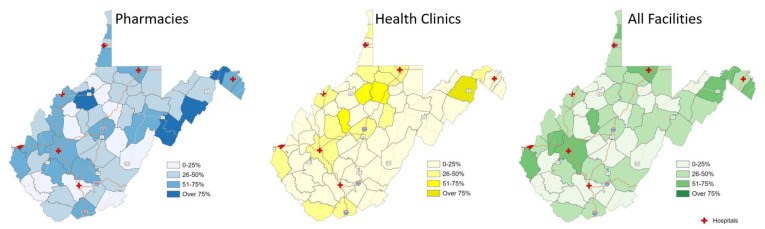
County distribution of Plan B reported availability by site type NOTE: Health clinic data were previously unpublished; pharmacy data used for mapping and methodology for health clinic data collection are described in Ashcraft et al., 2020.^15^

**Figure 3:**
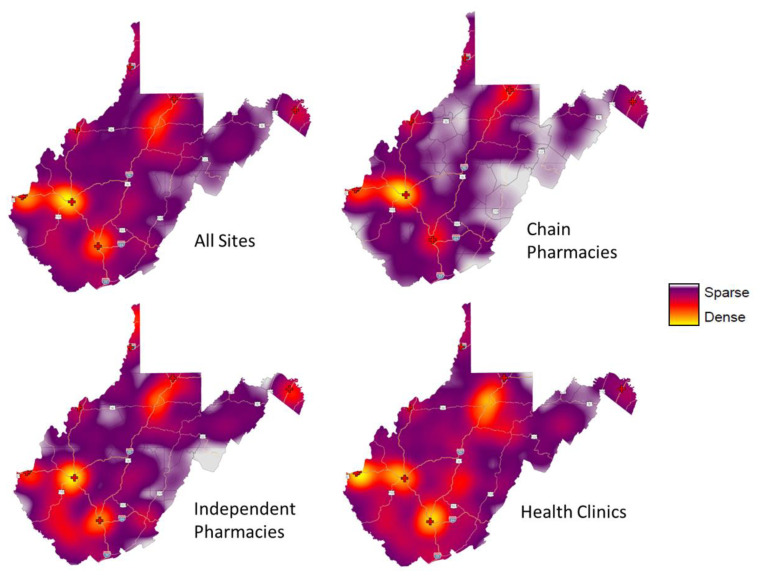
Site density by type NOTE: Health clinic data were previously unpublished; pharmacy data used for mapping and methodology for health clinic data collection are described in Ashcraft et al., 2020.^15^

**Figure 4:**
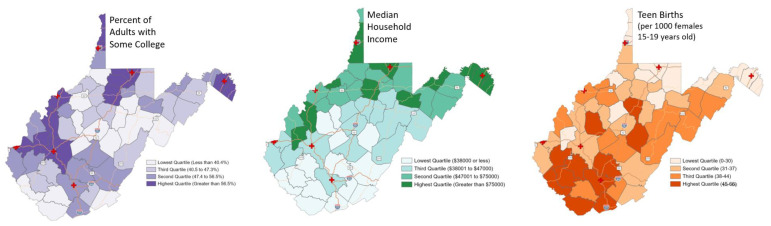
Socioeconomic demographic characteristics by WV county NOTE: Education and income data from DHHS^50^, teen birth data from Khan et al., 2022.^51^

Fig. 1 displays pharmacies and health clinics in WV by site type and their reported availability of LNG EC. Chain pharmacies are clearly clustered in the more populated areas of the state where major roads intersect. There are significant sections of the state with no nearby pharmacy or health clinic, and there are even larger portions of the state—whole counties—with no Plan B® available. Fig. 2 displays this data differently by using percent quartiles to illustrate reported LNG EC availability by site category, specifically pharmacies (blue), health clinics (yellow), and overall (green) in each county. Fig. 3 illustrates the density of pharmacies and health clinics across the state. Taken together, Figures 1–3 illustrate the extent of the contraceptive desert^49^ in WV. Finally, Fig. 4 presents WV county comparisons of sociodemographic factors from census data, including median household income and the percentage of adults with some college education,^50^ as well as teen pregnancy rates.^51^ The more northern and western counties have higher levels of education and income, while the most rural southern and southeastern counties have the highest rates of teen pregnancy. When these variables were used in models to predict LNG EC availability at sites, sociodemographic variables were able to explain about 26% of the variation in LNG EC availability (R^2^ = 0.26).

Access to LNG EC in WV was limited well before the decision on *Dobbs v. Jackson Women’s Health Organization*, but now it is getting worse. More than 1.5 million Appalachian women live in a contraceptive desert.^49^ In Central Appalachia, which includes parts of WV, Kentucky, Virginia, and Tennessee, 59.7% of women live where they do not have access to all contraceptive options.^49^

### Where Do We Go from Here?

In a rural region already plagued by poverty, chronic disease, substance use disorder and high rates of children in foster care, women are increasingly losing autonomy over their reproduction. The *Dobbs* decision prompted abortion trigger ban laws, gave rise to new state legislation to ban abortions, and reignited anti-abortion activists’ and legislators’ calls for a ban on EC. Widespread misperception about the mechanism of action of EC puts the medication at risk of further restriction or ban, particularly in rural Appalachia. Furthermore, the loss of constitutional protection of reproductive rights, in general, may allow individual pharmacies to feel justified in pulling LNG EC from the shelves. Women may soon need to travel out of state to access care for managing contraception, unplanned pregnancy, and abnormal pregnancy. The limiting of telemedicine options and insurance coverage out of state will make accessing appropriate care nearly impossible, especially for low-income patients.

Given that access to contraception has improved women’s educational attainment, increased labor force participation, improved career outcomes, decreased the likelihood of living in poverty, increased emotional and economic investment in their children, and improved economic well-being of children into adulthood, ensuring timely availability of all contraception is critical.^32^–^33^ With further restrictions to women’s reproductive rights on the horizon, specific legislation is needed to protect access to all contraception, including EC. It is essential to the health of Appalachia for women to maintain control over their own reproduction.

## Figures and Tables

**Table 1:** Type of WV health clinic contacted (n = 404)*

Type of Health Clinic	n (%)†
Federally Qualified Health Center	222 (55%)
County Health Department	51 (12.6%)
Rural Health Clinic	251 (62.1%)
Title X Clinic	124 (30.7%)
Free/Charitable Clinic	20 (5%)
College/University Health Center	16 (4%)

NOTES:

* These data were previously unpublished, but the methodology for data collection was described in detail in Ashcraft et al. 2020.^15^

† Proportions add up to more than 100% because some health centers are included in multiple categories.

**Table 2:** LNG EC availability by WV health clinic type (n = 404)*

Type of Health Clinic	Available today?			
	Yes	No	Unsure	Missing†
Federally Qualified Health Center	38 (17.1%)	57 (25.7%)	12 (5.4%)	115 (51.8%)
County Health Department	24 (47.1%)	11 (21.6%)	0 (0%)	16 (31.4%)
Rural Health Clinic	45 (17.9%)	70 (27.9%)	9 (3.6%)	127 (50.6%)
Title X Clinic	54 (43.5%)	17 (13.7%)	5 (4.0%)	48 (38.7%)
Free/Charitable Clinic	2 (10%)	4 (20%)	0 (0%)	14 (70%)
College/University Health Center	4 (25%)	5 (31.3%)	0 (0%)	7 (43.8%)

NOTES:

* These data were previously unpublished, but the methodology for data collection was described in detail in Ashcraft et al. 2020.^15^

† Data were missing for a variety of reasons, including unanswered phone calls, limited hours of availability, clinic closure, or the staff member we needed to speak with was not available on two separate call attempts placed Monday – Friday, 9 a.m. – 5 p.m.
